# Pediatric mortality from neurofibromatosis and malignant peripheral nerve sheath tumors in Brazil, 2008–2023: a 16-year nationwide analysis of persistent disparities

**DOI:** 10.1007/s00381-026-07415-5

**Published:** 2026-07-24

**Authors:** Karina Munhoz de Paula Alves Coelho, José Guilherme Pickler, José Cândido Caldeira Xavier-Júnior, Francis Rossetti Pedack, Raquel Francine Liermann Garcia, Carlos Frederico Fronza, Bruna Louise Silva, Acir Alves Coelho Junior, Paulo Henrique Condeixa de França, Bárbara Sarni Sanches, Priscila Ferraz Franczak, Gustavo Rassier Isolan, Marcelo Gerardin Poirot Land, Rafael Roesler

**Affiliations:** 1 Department of Scientific Development and Innovation (DECIPE), Center for Anatomo-Pathological Diagnosis (CEDAP), Joinville, Brazil; 2 National Science and Technology Institute for Children’s Cancer Biology and Pediatric Oncology-INCT BioOncoPed, Porto Alegre, Brazil; 3 Graduate Program in Medical Sciences, Faculty of Medicine, Federal University of Rio Grande Do Sul, Porto Alegre, Brazil; 4 Graduate Program in Health and Environmental Sciences, University of the Joinville Region (UNIVILLE), Joinville, Brazil; 5 School of Medicine, Unisalesiano Catholic University Center, Araçatuba, SP Brazil; 6 Araçatuba Institute of Pathology, Araçatuba, Brazil; 7 Graduate Program in Pathology, School of Medicine, São Paulo State University (UNESP), Botucatu, Brazil; 8 Department of Surgery, São José Municipal Hospital, Joinville, Brazil; 9 Department of Mechanical Engineering, Center for Technological Sciences, Santa Catarina State University (UDESC), Joinville, Brazil; 10 Graduate Program in Internal Medicine, Faculty of Medicine, Federal University of Rio de Janeiro (UFRJ), Rio de Janeiro, Brazil; 11 Department of Software Engineering, University of the Joinville Region (UNIVILLE), Joinville, Brazil; 12 Graduate Program in Principles of Surgery, Mackenzie Evangelical University, Curitiba, Brazil; 13 The Center for Advanced Neurology and Neurosurgery (CEANNE), Porto Alegre, Brazil; 14 Martagão Gesteira Institute of Childcare and Pediatrics (IPPMG-UFRJ), Graduate Program in Pediatric and Maternal Health, Federal University of Rio de Janeiro, Rio de Janeiro, Brazil; 15 Department of Pharmacology, Institute for Basic Health Sciences, Federal University of Rio Grande Do Sul, Porto Alegre, Brazil; 16 Cancer and Neurobiology Laboratory, Experimental Research Center, Clinical Hospital (CPE-HCPA), Federal University of Rio Grande Do Sul, Porto Alegre, Brazil; 17 Center for Biotechnology, Federal University of Rio Grande Do Sul, Porto Alegre, Brazil

**Keywords:** Neurofibromatosis 1, Nerve sheath neoplasms, Pediatric mortality, Pediatric oncology, Epidemiology, Healthcare disparities

## Abstract

**Purpose:**

Neurofibromatosis (NF) comprises genetic tumor predisposition syndromes with multisystem involvement, yet pediatric mortality data remain scarce in middle-income settings. Malignant peripheral nerve sheath tumors (MPNST) represent the leading cause of premature death in this population. This study analyzed 16-year temporal trends and regional disparities in pediatric NF/MPNST mortality and hospitalizations in Brazil.

**Methods:**

We conducted a nationwide ecological study of individuals aged 0–19 years from 2008 to 2023. Mortality data were obtained from the Mortality Information System (SIM) and hospital admissions from the Hospital Information System (SIH/SUS), with temporal trends assessed using Prais–Winsten regression. We calculated Age-Specific Mortality Rates (ASMRs) and assessed excess mortality risk using Standardized Mortality Ratios (SMR) to identify regional disparities.

**Results:**

A total of 177 pediatric deaths were identified, with MPNST accounting for 64.9% (*N* = 115) and NF for 35.1% (*N* = 62). SMR analysis revealed significant geographic inequalities. Children aged 0–9 in the South region faced a mortality risk double that of the reference population (SMR = 2.02; 95% CI 1.08–3.46; *p* = 0.010), whereas adolescents in the North region exhibited significantly lower-than-expected mortality. Despite global therapeutic advances, mortality rates in Brazil remained statistically stagnant across all age groups and conditions (*p* values were non-significant). Conversely, NF-related hospitalizations demonstrated an increasing trend (+ 2.98% annually for ages 0–9; + 1.94% for ages 10–19), while MPNST admissions remained stable.

**Conclusion:**

Pediatric mortality from NF and MPNST in Brazil has remained unchanged for 16 years, contrasting with the rising trend in NF-related hospitalizations. This discrepancy, coupled with marked regional disparities, suggests persistent structural challenges in early diagnosis and equitable access to specialized oncology care. These findings highlight a critical need for national notification systems and improved therapeutic strategies for affected children and adolescents.

## Introduction

Neurofibromatosis (NF) comprises a heterogeneous group of autosomal dominant neurocutaneous syndromes characterized by germline tumor predisposition, primarily affecting the nervous and cutaneous systems. Neurofibromatosis type 1 (NF1) is the most common form, occurring in 1 in 2,000–3,000 births, and is defined by café-au-lait macules, axillary freckling, neurofibromas, Lisch nodules, optic pathway gliomas, and skeletal abnormalities [1, 2]. Neurofibromatosis type 2 (NF2) typically presents with bilateral vestibular schwannomas and meningiomas, whereas schwannomatosis, the rarest subtype, features multiple non-vestibular schwannomas frequently associated with chronic pain [3, 4].

Advances in genomic research have clarified the molecular basis of these disorders. NF1 results from pathogenic variants in the *NF1* gene on chromosome 17q11.2, leading to loss of neurofibromin and dysregulation of the *RAS/MAPK* pathway [5]. NF2 arises from loss-of-function variants in *NF2* on chromosome 22q12, encoding merlin, a tumor suppressor that regulates *PI3K/AKT, Raf/MEK/ERK*, and mTOR signaling [6]. Updated classifications now define NF2 as “NF2-related schwannomatosis,” while pathogenic variants in *SMARCB1* and *LZTR1* account for most schwannomatosis cases [7–9]. Genotype–phenotype studies highlight substantial clinical variability, underscoring the need for personalized surveillance and management strategies [10–12].

In children and adolescents, NF contributes to significant morbidity, including gliomas, plexiform neurofibromas, skeletal abnormalities, and learning disabilities. Malignant peripheral nerve sheath tumor (MPNST) is the leading cause of premature mortality in this population [13–15]. Individuals with NF1 present an increased risk of developing MPNST due to NF1 gene inactivation and consequent loss of neurofibromin function, leading to dysregulation of the RAS/MAPK signaling pathway and favoring malignant transformation [5]. MPNST represents a major oncologic complication of NF1, with an estimated lifetime risk of 8–13%; notably, nearly half of all MPNSTs arise in individuals with NF1 [16, 17]. Despite advances in molecular diagnostics and targeted therapies, it remains unclear whether these developments have translated into measurable reductions in pediatric mortality at the population level.

European studies from Finland, France, and Italy have documented increased mortality in NF1 [18–20], yet the Italian investigation from 1995–2006 did not specifically evaluate pediatric outcomes [21]. While additional research from high-income nations has described NF1 mortality [22–24], evidence from large, ethnically diverse, upper-middle-income countries remains scarce [25–27].

Brazil, the largest upper-middle-income country in Latin America, with more than 215 million inhabitants and a universal public healthcare system (Sistema Único de Saúde – SUS), offers a distinct epidemiological context for examining these patterns. Here, we address a critical knowledge gap by analyzing 16 years of nationwide data to characterize temporal trends and regional disparities in NF/MPNST mortality and hospitalizations among children and adolescents outside the traditional North American and European settings.

## Methods

### Study design and data sources

We conducted a nationwide, retrospective ecological study, in which the unit of analysis was the population-level aggregate, to analyze pediatric mortality and hospitalizations associated with NF (without stratification between NF1 and NF2) and MPNST in Brazil over a 16-year period (2008–2023). This study adheres to the Strengthening the Reporting of Observational Studies in Epidemiology (STROBE) guidelines, adapted for ecological study designs.

Mortality data were extracted from the Mortality Information System (*Sistema de Informações sobre Mortalidade* – SIM), and hospitalization data were obtained from the Hospital Information System (*Sistema de Informações Hospitalares* – SIH/SUS). Both databases are managed by the Department of Informatics of the Unified Health System (DATASUS). Age was stratified into four groups (0–4, 5–9, 10–14, and 15–19 years) according to DATASUS pediatric age categories. Demographic data, used as denominators for rate calculations, were obtained from the Brazilian Institute of Geography and Statistics (*Instituto Brasileiro de Geografia e Estatística* – IBGE), utilizing census data and official intercensal estimates. All data extraction was performed in September 2025.

This study utilized publicly available, anonymized secondary data from DATASUS and IBGE. In accordance with Brazilian regulations (Resolution 466/2012 of the National Health Council), this study, based exclusively on anonymized secondary data, was classified as minimal risk and exempt from formal ethics committee approval and individual informed consent.

### Case definition and study variables

The study population included pediatric individuals aged 0 to 19 years. Cases were identified using the International Classification of Diseases, 10th Revision (ICD-10). Inclusion criteria comprised ICD-10 code Q85.0 for NF. Given the absence of a specific ICD-10 code for MPNST, cases were identified using codes C47.0–C47.9, which encompass malignant neoplasms of peripheral nerves and are commonly used as a proxy for MPNST in population-based studies. We excluded records coded as tuberous sclerosis (Q85.1), other specified phakomatoses (Q85.8), and unspecified phakomatosis (Q85.9) to ensure specificity.

Extracted variables included year of death, age, sex, state of residence, and causes of death listed in Lines A–D of the death certificate. For spatial analysis, the 26 Brazilian states and the Federal District were aggregated into five macroregions: North, Northeast, Southeast, South, and Midwest. Age was stratified into four groups: 0–4, 5–9, 10–14, and 15–19 years.

### Statistical analysis

#### Descriptive and comparative metrics

Descriptive statistical analysis was conducted to summarize the absolute number of deaths by year, age group, sex, and geographic region. The proportion of deaths attributed to NF and MPNST was also calculated. Patterns in mortality distribution were examined across time, demographic strata, and geographic areas.

#### Standardized mortality ratio (SMR)

To quantify excess mortality relative to expected levels, we calculated the standardized mortality ratio (SMR) (SMR = O/E), where O represents observed deaths and E represents expected deaths. For age-specific SMR, expected deaths were calculated by applying age-specific reference rates to the exposed population: (${E}_{age}={r}_{age}^{ref}\times {P}_{age}$). For macro-regional estimates without age stratification, the aggregate national reference rate was applied: (${E}_{macro}={r}_{total}^{ref}\times {P}_{macro}$).

Confidence intervals (CI) of 95% for the SMR were determined based on sample size. For strata with small counts (O < 15), we used the exact interval based on the Poisson distribution. For strata with larger counts (O ≥ 15), we employed the Byar asymptotic approximation, which provides reliable estimates for moderate to large event counts.

#### Temporal trend analysis

Rates were calculated separately for the 0–9 and 10–19 year age groups, using the corresponding annual population estimate for each age group and calendar year as the denominator. Because annual population estimates were used throughout the study period, temporal changes in the size and age composition of the population were incorporated into the rate calculations. No direct or indirect age standardization using a fixed reference population was performed, as the analyses focused on age-specific trends within each age group.

Temporal trends in mortality and hospitalization rates were evaluated for two age clusters (0–9 and 10–19 years) using Prais–Winsten regression, a method selected to correct for first-order serial autocorrelation inherent in time-series data.

From these models, we calculated the Annual Percent Change (APC) with a 95% CI. Trends were classified as *increasing*, *decreasing*, or *stable* according to the direction and statistical significance of the APC (95% CI). Results were visualized by plotting observed annual rates overlaid with the fitted regression lines and 95% confidence bands.

The analysis was conducted in two stages. First, a descriptive assessment of pediatric mortality (0–19 years) associated with NF and MPNST was performed to characterize demographic and age-specific patterns. This was followed by a comparative assessment of mortality risk using SMR to identify regional disparities, and an evaluation of temporal trends in mortality and hospitalization rates using Prais–Winsten regression.

## Results

### Descriptive statistical analysis

**Fig. 1:**
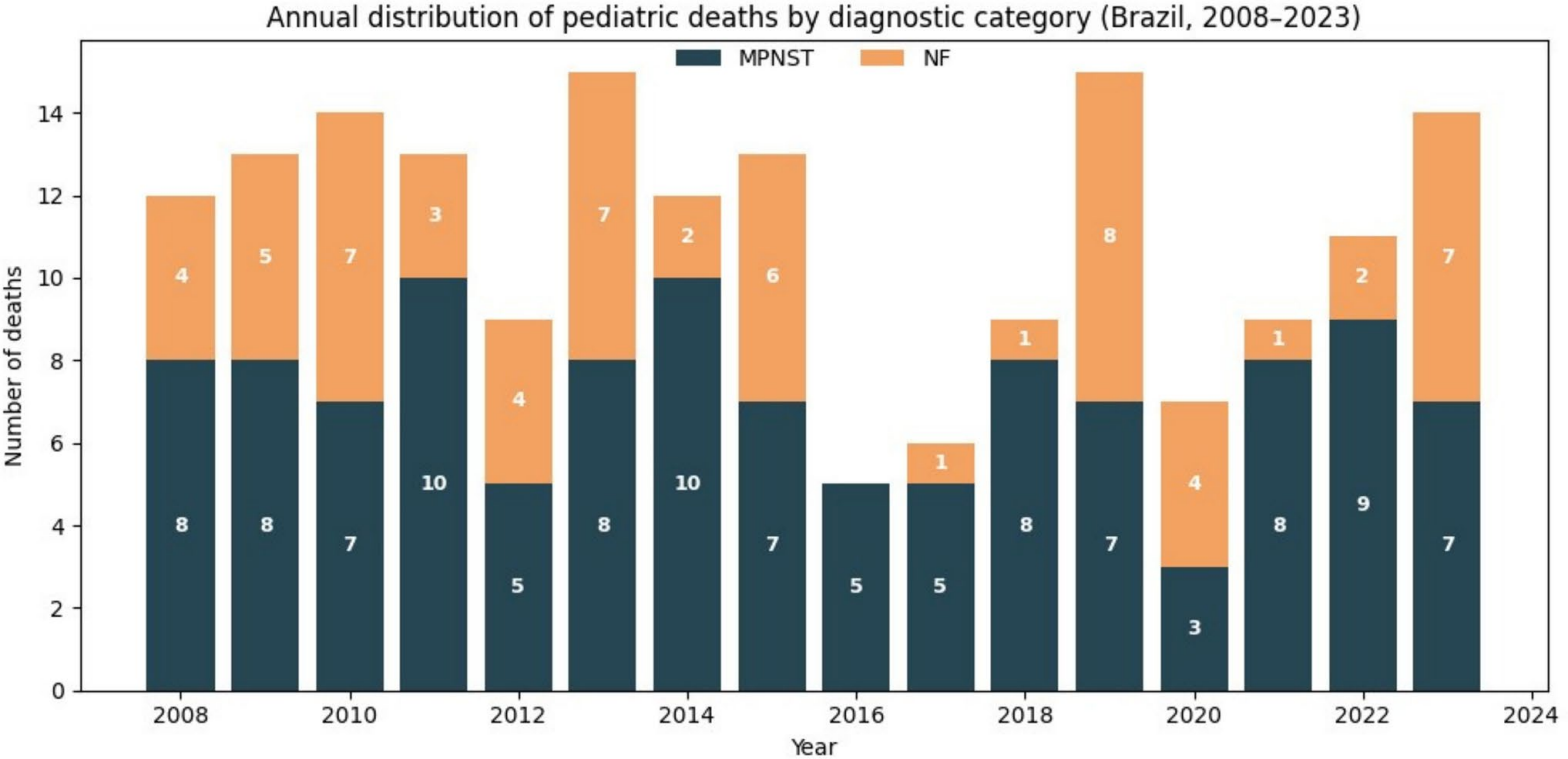
Annual distribution of pediatric deaths (0–19 years) associated with neurofibromatosis (NF) and malignant peripheral nerve sheath tumors (MPNST) in Brazil, 2008–2023. Stacked bars represent the absolute number of deaths per year according to diagnostic category. Over the 16-year period, MPNST accounted for the majority of deaths (64.9%), while NF represented 35.1%. Although year-to-year fluctuations were observed, no consistent declining pattern was evident over time

Between 2008 and 2023, a total of 177 pediatric deaths attributable to NF and MPNST were recorded in Brazil. Most deaths were associated with MPNST, which accounted for 64.9% (*n* = 115) of the total, while NF represented 35.1% (*n* = 62). As illustrated in Fig. 1, the annual number of deaths showed fluctuation throughout the study period, with peaks observed in 2013 (*n* = 15) and 2019 (*n* = 15).

**Fig. 2:**
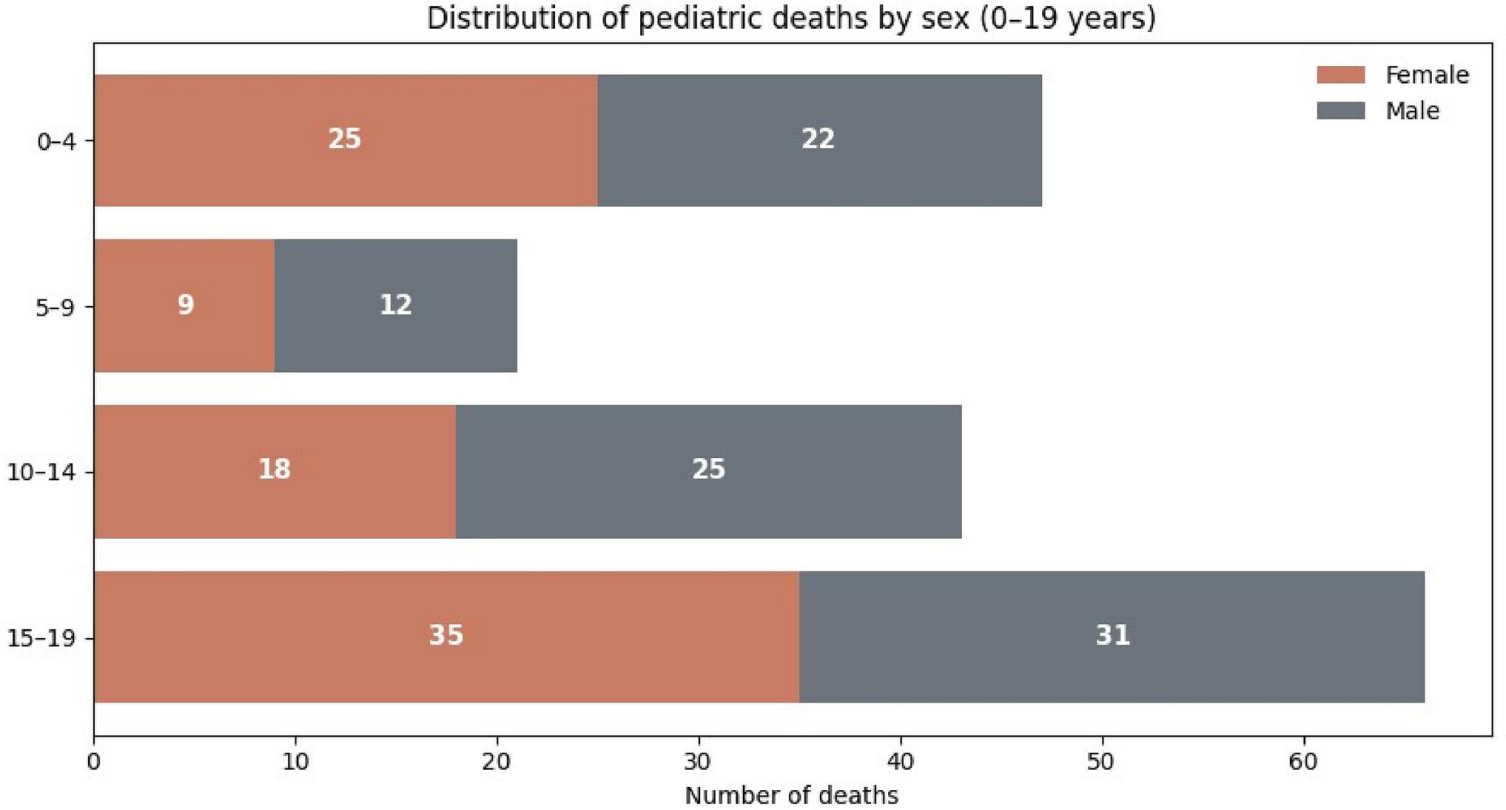
Distribution of pediatric deaths (0–19 years) associated with neurofibromatosis and malignant peripheral nerve sheath tumors in Brazil (2008–2023), according to sex and age group. Horizontal stacked bars represent the absolute number of deaths within each age stratum. Overall mortality was relatively balanced between females and males; however, sex distribution varied across age groups. Females accounted for more deaths in early childhood (0–4 years) and late adolescence (15–19 years), whereas males predominated in the intermediate age groups (5–9 and 10–14 years), highlighting age-dependent variations in sex-specific mortality patterns

Analysis of mortality by age group revealed a distinct bimodal distribution. The highest concentration of deaths occurred in late adolescence (15–19 years, n = 66, 37.3%), followed by early childhood (0–4 years, n = 47, 26.6%). The overall sex distribution was relatively balanced (50.9% males vs. 49.1% females). However, this balance shifted across age strata: females experienced higher mortality in the 0–4 and 15–19 age groups, whereas males accounted for more deaths in the 5–9 and 10–14 brackets (Fig. 2).

**Fig. 3:**
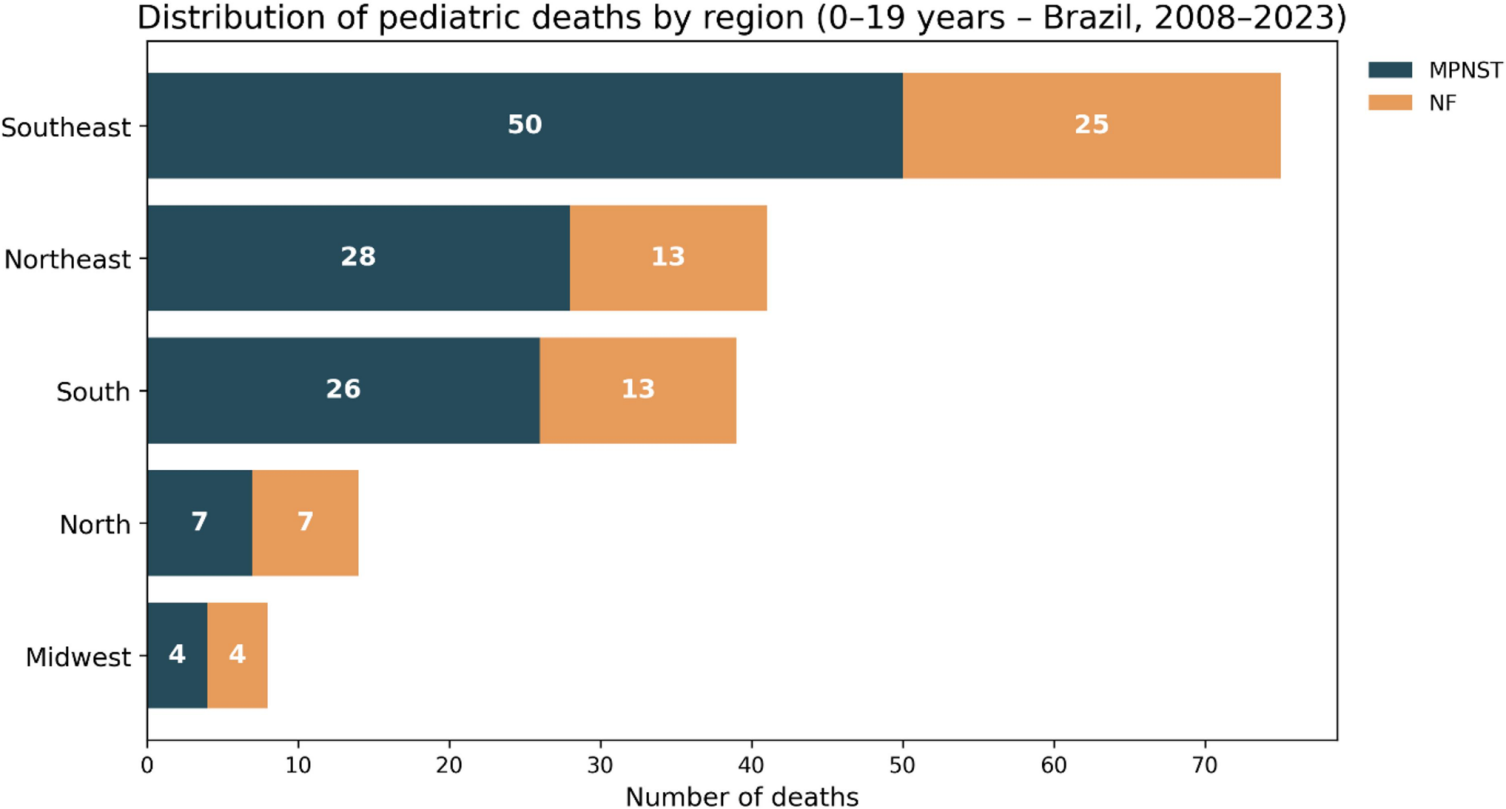
Distribution of pediatric deaths (0–19 years) by geographic macro-region in Brazil (2008–2023)

In terms of geographic distribution, the absolute number of deaths was highest in the Southeast region, reflecting the population density of the area, followed by the Northeast and South regions (Fig. 3).

### Comparative mortality risk (SMR) and regional disparities

To evaluate regional and age-specific disparities, observed deaths were compared to expected deaths based on the standard Brazilian pediatric population, yielding the Standardized Mortality Ratio (SMR). This analysis revealed significant geographic heterogeneities, particularly for malignant cases (Table 1).

**Table 1:** Comparative analysis of observed versus expected pediatric deaths (standardized mortality ratio (SMR)) for neurofibromatosis (NF) and malignant peripheral nerve sheath tumors (MPNST) by age group and region, Brazil (2008–2023)

Condition	Age group	Region	Observed	Expected	SMR (95% CI)	*p*-value
MPNST	0–9 years	South	13	6.43	2.02 (1.08–3.46)	0.010
		Southeast	13	18.98	0.68 (0.36–1.17)	0.170
		Northeast	14	14.29	0.98 (0.54–1.64)	0.938
		North	6	5.38	1.12 (0.41–2.43)	0.789
		Midwest	3	3.92	0.77 (0.16–2.24)	0.643
	10–19 years	Southeast	37	25.77	1.44 (1.01–1.98)	0.027
		South	13	8.84	1.47 (0.78–2.52)	0.161
		Northeast	14	19.44	0.72 (0.39–1.21)	0.217
		North	1	6.85	0.15 (0.00–0.81)	0.025
		Midwest	1	5.10	0.20 (0.00–1.09)	0.069
NF	0–9 years	South	4	2.49	1.60 (0.44–4.11)	0.340
		Southeast	8	7.36	1.09 (0.47–2.14)	0.814
		Northeast	4	5.54	0.72 (0.20–1.85)	0.512
		North	1	2.08	0.48 (0.01–2.67)	0.452
		Midwest	2	1.52	1.32 (0.16–4.76)	0.696
	10–19 years	South	9	5.76	1.56 (0.71–2.97)	0.177
		Southeast	17	16.79	1.01 (0.59–1.62)	0.959
		Northeast	9	12.67	0.71 (0.32–1.35)	0.303
		North	6	4.46	1.34 (0.49–2.93)	0.467
		Midwest	2	3.32	0.60 (0.07–2.17)	0.468

95% CI calculated using the exact Poisson method for O < 15 and Byar approximation for O ≥ 15; *p*-value derived from Wald test (approximate)

*SMR* standardized mortality ratio, *CI* confidence interval

For MPNST, children aged 0–9 years in the South region presented a mortality risk more than twice the expected for the reference population (SMR = 2.02; 95% CI 1.08–3.46; *p* = 0.010). Similarly, in the Southeast region, adolescents aged 10–19 years exhibited a 44% higher risk of mortality (SMR = 1.44; 95% CI 1.01–1.98; *p* = 0.027). Conversely, adolescents in the North region showed a statistically significant reduction in SMR (SMR = 0.15; 95% CI 0.00–0.81; *p* = 0.025). This finding may reflect regional differences in diagnostic capacity or case ascertainment.

For NF, no statistically significant deviations in SMR were observed across any age group or geographic region (*p* > 0.05 for all strata). However, the consistently wide confidence intervals suggest that the lack of statistical significance may be influenced by the smaller number of events (*N* = 62) limiting the statistical power to detect regional variations, rather than a definitive absence of risk differences.

### Temporal trends in mortality and hospitalizations

**Fig. 4:**
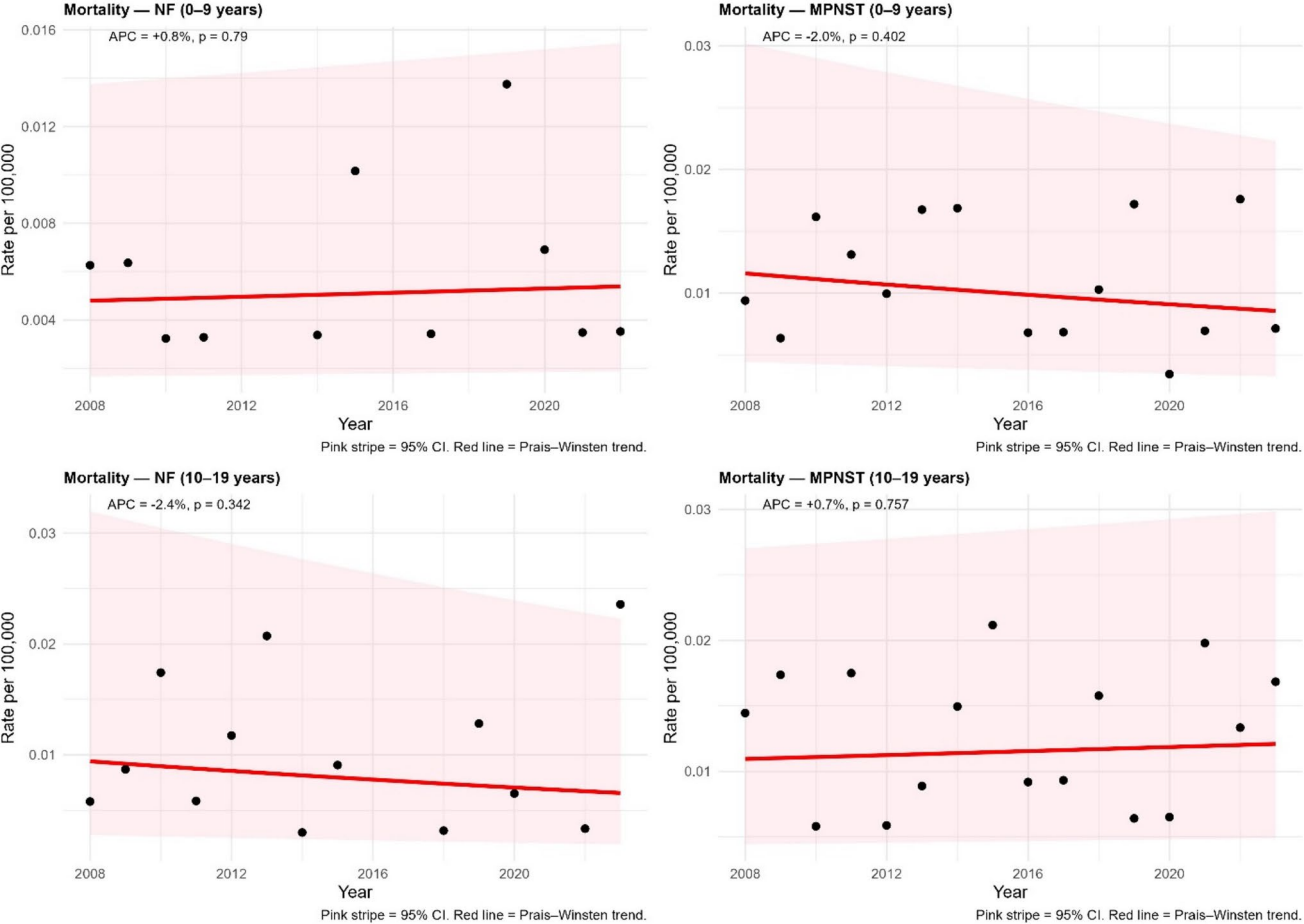
Temporal trends in pediatric mortality rates (per 100,000 population) associated with neurofibromatosis (NF) and malignant peripheral nerve sheath tumors (MPNST) from 2008 to 2023

**Fig. 5:**
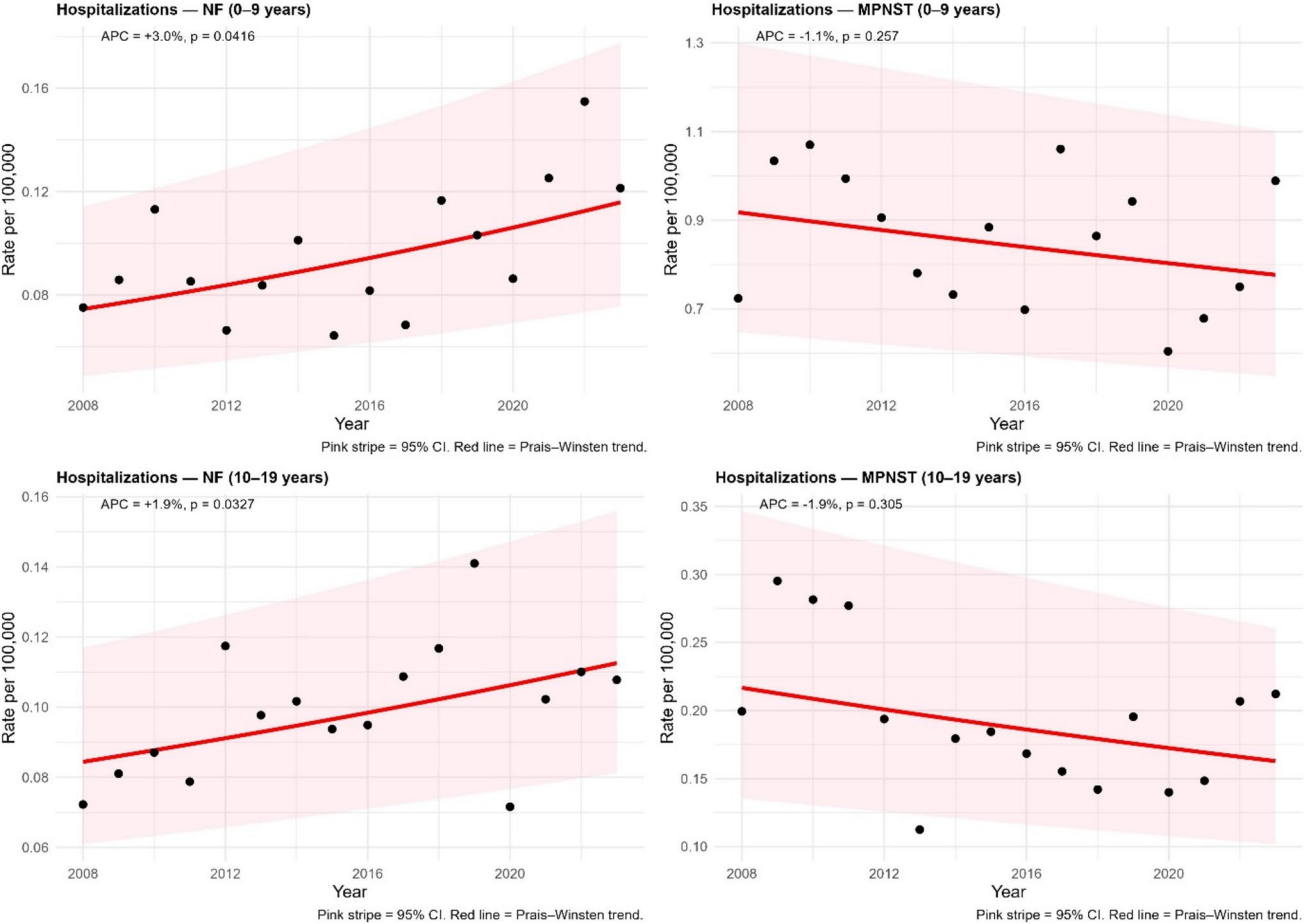
Temporal trends in pediatric hospitalizations associated with neurofibromatosis (NF) and malignant peripheral nerve sheath tumors (MPNST) from 2008 to 2023

The Prais–Winsten regression for temporal trend analysis revealed distinct patterns between mortality and hospital admissions over the 16-year period (Figs. 4 and 5). Mortality rates remained stable across all analyzed strata. No statistically significant upward or downward trends were observed for either NF or MPNST in any age group. For instance, MPNST mortality in the 0–9 age group showed an Annual Percent Change (APC) of −2.00% (*p* = 0.402), while NF mortality in adolescents (10–19 years) showed an APC of −2.39% (*p* = 0.342), confirming the stability of lethal outcomes (Table 2).

**Table 2:** Temporal trend analysis (Prais–Winsten regression) for pediatric mortality and hospitalization rates associated with neurofibromatosis (NF) and malignant peripheral nerve sheath tumors (MPNST) in Brazil (2008–2023)

Age range	Type	Outcome	APC (%)	95% CI	*p*-value	Trend
0–9 years	NF	Hospitalizations	+ 2.98	0.13 to 5.91	0.0416	Increasing
	MPNST	Hospitalizations	− 1.11	− 3.08 to 0.91	0.257	Stable
	NF	Mortality	+ 0.83	− 5.84 to 7.98	0.790	Stable
	MPNST	Mortality	− 2.00	− 6.82 to 3.06	0.402	Stable
10–19 years	NF	Hospitalizations	+ 1.94	0.18 to 3.72	0.0327	Increasing
	MPNST	Hospitalizations	− 1.89	− 5.58 to 1.95	0.305	Stable
	NF	Mortality	− 2.39	− 7.47 to 2.97	0.342	Stable
	MPNST	Mortality	+ 0.67	− 3.79 to 5.33	0.757	Stable

Statistical significance set at *p* < 0.05.

*APC* annual percent change, *CI* confidence interval

Hospitalization trends, however, diverged significantly by diagnosis. While MPNST-related hospitalizations remained stable over time, NF-related hospitalizations exhibited a statistically significant increasing trend. In children aged 0–9 years, NF hospitalizations increased by an average of 2.98% per year (*p* = 0.0416). A similar upward trajectory was observed in the 10–19 age group, with an annual increase of 1.94% (*p* = 0.0327). Despite advances in molecular diagnostics and clinical management during the study period, no measurable reduction in pediatric mortality was observed at the population level.

## Discussion

The most critical finding of this study is the persistence of pediatric mortality associated with NF and MPNST in Brazil over a 16-year period, with no evidence of decline. This finding is particularly concerning when contrasted with global pediatric oncology trends, in which mortality from most childhood cancers has declined in high-income settings following advances in therapy and supportive care [28]. Mortality was more frequently observed among adolescents aged 15 to 19 years, underscoring an ongoing public health concern in this population.

The age-specific mortality distribution associated with NF/MPNST exhibited a clear bimodal pattern, with peaks in early childhood and late adolescence, without consistent differences between sexes. Although MPNST accounted for more than 60% of the recorded deaths, this finding should be interpreted in light of the difficulty in identifying patients with underlying NF prior to malignant transformation, a bias well documented in population-based registry studies in which mild or unrecognized cases tend to be underdiagnosed [29]. Benign manifestations of NF, such as cutaneous neurofibromas, may be clinically mistaken for other non-malignant lesions and, because they are generally considered benign, are not routinely submitted for histopathological evaluation. This may contribute to delayed recognition of the disease and potential underidentification of NF-related deaths in administrative databases [29]. In this context, the establishment of structured national notification systems or disease registries for NF could enhance case ascertainment and provide a more accurate estimation of mortality patterns at the population level.

In the analysis of hospital admissions related to NF and MPNST, distinct temporal patterns were observed between the two conditions. While hospitalization trends for MPNST remained stable over time, NF-related hospitalizations showed a statistically significant increasing trend in both pediatric age groups analyzed. These patterns may reflect multiple factors, including changes in healthcare utilization, diagnostic practices, access to care, and coding practices over time. However, the available data do not allow determination of the specific drivers underlying the observed increase in NF-related hospitalizations [30]. Ecological studies addressing NF- and MPNST-related outcomes are relatively scarce, and most available investigations focus exclusively on NF1 and are conducted in high-income countries. An Italian study and a North American study reported higher frequencies of NF1-related deaths than those observed in the present analysis [21, 24]. The IDEMA French study corroborated the concentration of deaths during the second decade of life and reported higher female mortality in this age group, partially consistent with our findings, in which females accounted for more deaths in the 15–19 age group, although without a consistent female predominance across all age strata [22]. Comparisons across studies remain challenging due to differences in study periods, age stratification, and data sources [21–23]. Previous Brazilian studies have also highlighted the substantial clinical burden and epidemiological heterogeneity of NF1, reinforcing the importance of long-term surveillance and healthcare strategies for affected patients in the national setting [31, 32]. These limitations highlight the relevance of the present study in providing a nationwide perspective on NF and MPNST mortality in a middle-income country. Additionally, the year-to-year oscillations observed in mortality rates should be interpreted cautiously, as they likely reflect the low absolute number of annual events and the expected variability inherent to rare diseases in population-based analyses.

This study has several limitations inherent to the use of administrative databases, including potential misclassification, incomplete clinical information, and the inability to assess disease severity, treatment strategies, or long-term outcomes. The use of ICD-10 code C47 as a proxy for MPNST may have introduced misclassification, as this code encompasses malignant neoplasms of peripheral nerves not exclusively attributable to NF. Although the magnitude of this potential misclassification bias cannot be determined from the available data, the inclusion of other malignant tumors of peripheral nerves and the autonomic nervous system classified under ICD-10 code C47 may have resulted in a slight overestimation of MPNST-related mortality and hospitalization rates. In addition, hospitalization data capture only inpatient care and do not reflect outpatient management, which constitutes a substantial component of NF care. Similar methodological challenges have been reported in studies conducted in high-income countries [21]. The French study differs in this regard, as it analyzed cases from a National Referral Center for Neurofibromatoses rather than a population-based administrative database [22]. Furthermore, it was not possible to identify probands, family clusters, or specific NF subtypes in the present study. Despite these limitations, this analysis provides valuable nationwide insights into mortality and hospitalization patterns associated with NF and MPNST in Brazil.

## Conclusion

In the absence of measurable reductions in pediatric mortality over more than a decade, coordinated national strategies for early detection of malignant transformation and timely referral to specialized centers may be critical to improving outcomes. Future studies should aim to integrate hospitalization, mortality, and outpatient datasets to better estimate the full burden of NF and MPNST. Multicenter clinical cohorts may further clarify prognostic factors, outcomes, and healthcare utilization. In addition, digital health strategies may support earlier identification and longitudinal monitoring of patients with NF within the SUS, potentially improving equity in access and outcomes.

## Data Availability

The datasets analyzed during the current study are publicly available from the DATASUS platform (SIM and SIH/SUS) and from the Brazilian Institute of Geography and Statistics (IBGE). All data are publicly accessible and do not require individual-level authorization.
